# Plant terpenes: defense responses, phylogenetic analysis, regulation and clinical applications

**DOI:** 10.1007/s13205-014-0220-2

**Published:** 2014-04-29

**Authors:** Bharat Singh, Ram A. Sharma

**Affiliations:** 1 AIB, Amity University Rajasthan, NH-11C, Kant Kalwar, Jaipur, 303 002 India; 2 Department of Botany, University of Rajasthan, Jaipur, 302 055 India

**Keywords:** Terpenes, Terpene synthase, Phylogenetic analysis, Clinical trials

## Abstract

The terpenoids constitute the largest class of natural products and many interesting products are extensively applied in the industrial sector as flavors, fragrances, spices and are also used in perfumery and cosmetics. Many terpenoids have biological activities and also used for medical purposes. In higher plants, the conventional acetate-mevalonic acid pathway operates mainly in the cytosol and mitochondria and synthesizes sterols, sesquiterpenes and ubiquinones mainly. In the plastid, the non-mevalonic acid pathway takes place and synthesizes hemi-, mono-, sesqui-, and diterpenes along with carotenoids and phytol tail of chlorophyll. In this review paper, recent developments in the biosynthesis of terpenoids, indepth description of terpene synthases and their phylogenetic analysis, regulation of terpene biosynthesis as well as updates of terpenes which have entered in the clinical studies are reviewed thoroughly.

## Introduction

Plants produce various types of secondary metabolites, many of which have been subsequently exploited by humans for their beneficial roles in a diverse array of biological functions (Balandrin et al. 1985). Several terpenoids have their roles in plant defense against biotic and abiotic stresses or they are treated as signal molecules to attract the insects of pollination. Out of the investigated terpenoids, many have pharmacological and biological activities and are, therefore, interesting for medicine and biotechnology. The first step of terpenoid biosynthesis is generation of C_5_ unit like as isopentenyl diphosphate (IPP) or dimethylallyl diphosphate (DMAPP). For this study, two different separate pathways have been investigated that can generate the C_5_ unit: the mevalonate and methylerythritol phosphate (MEP) pathway. On the basis of C_5_ units, we can classify the terpenoids as C_5_ (hemiterpenes), C_10_ (monoterpenes), C_15_ (sesquiterpenes), C_20_ (diterpenes), C_25_ (sesterpenes), C_30_ (triterpenes), C_40_ (tetraterpenes), >C_40_ (polyterpenes) (Ashour et al. 2010; Martin et al. 2003).

The terpene synthases are responsible for the synthesis of terpenes; they can easily acquire new catalytic properties by minor changes in the structures (Keeling et al. 2008). The synthesis of monoterpenes is initiated by dephosphorylation and ionization of geranyl diphosphate to geranyl carbocation (Huang et al. 2010) and the synthesis of sesquiterpene starts with the ionization of farnesyl diphosphate to farnesyl cation, which can also be isomerized to nerolidyl cation (Degenhardt et al. 2009). Diterpenes are synthesized by diterpene synthases in two different pathways: via the ionization of diphosphate, as catalyzed by class I enzyme and the other is via the substrate protonation at the 14, 15-double bond of geranyl geranyl diphosphate; reaction is catalyzed by class II enzymes (Tholl 2006). The nonsteroidal triterpenoids are produced by conversion of squalene into oxidosqualene and cyclization via formation of dammarenyl cation; reaction is catalyzed by oxidosqualene cyclases (Phillips et al. 2006). Many terpenoids also possess the pharmaceutical properties and currently are being used in clinical practices. Among these terpenoids, taxol (diterpene) of *Taxus buccata* and artemisinin (sesquiterpene lactone) from*Artemisia annua* are well known antineoplastic and antimalarial agents (Croteau et al. 2006; Pollier et al. 2011).

This review deals with biosynthesis of terpenoids, phylogeny of terpene synthases, regulation of terpene biosynthesis, and also about the studies of the clinical trials of terpenoids. This review highlights the current approaches of the phylogenetic analysis of terpene synthases and regulation of terpenoids.

## Biosynthesis of terpenoids

**Fig. 1:**
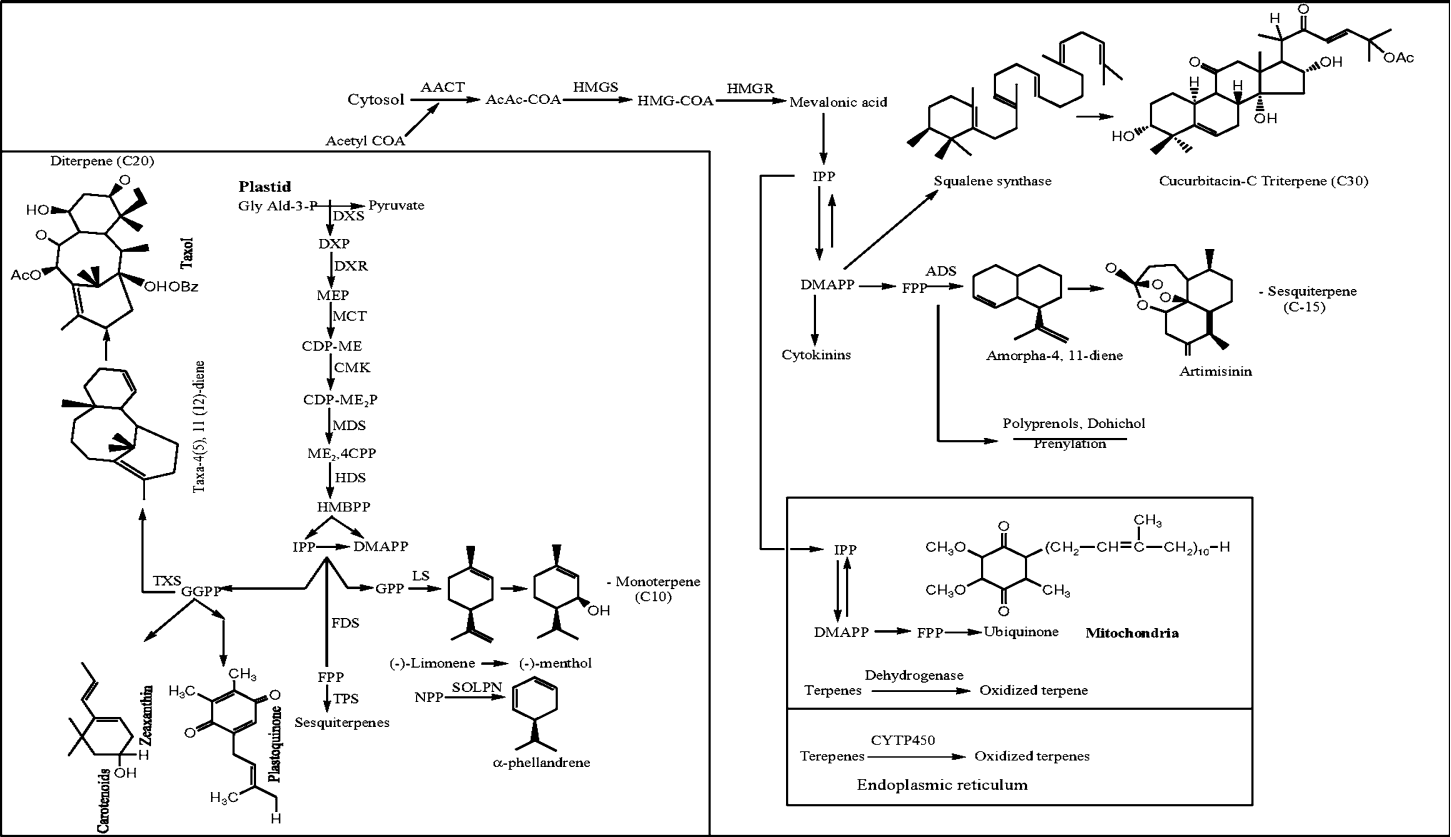
Schematic overview of monoterpenoid, sesquiterpenoid, diterpenoid and triterpenoid biosynthetic pathways. *AACT* acetoacetyl-CoA thiolase, *AcAc-CoA* acetoacetyl-CoA, *HMGS* HMG-CoA synthase, *HMG-CoA* 3-hydroxy-3-methylglutaryl, *HMGR* HMG-CoA-reductase, *IPP* isopentenyl diphosphate, *DMAPP* dimethylallyl diphosphate, *FPP* farnesyl pyrophosphate, *ADS* amorpha-4,11-diene synthase, *CYT450* cytochrome P_450_ hydroxylase, *GlyAld-3P* glyceraldehyde-3-phosphate, *DXP* deoxyxylulose-5-phosphate, *DXS* DXP synthase, *MEP* methylerythritol-4-phosphate, *DXR* DXP reductoisomerase, *CDP-OME* 4-(cytidine-5′-diphospho)-2-C-methyl-d-erythritol, *MCT* 2-C-methyl-d-erythritol-4-phosphate-cytidylyl transferase, *CDP-ME2P* 4-(cytidine-5′-diphospho)-2-C-methyl-d-erythritol phosphate, *CMK* CDP-ME Kinase, *ME2, 4cPP* 2-C-methyl-d-erythritol, 2,4-cyclodiphosphate, *MDS* 2-C-methyl-d-erythritol-2,4-cyclodiphosphate synthase, *HMBPP* (*E*)-4-hydroxy-3-methylbut-2-enyl diphosphate, *HDS* (*E*)-4-hydroxy-3-methylbut-2-enyl diphosphate synthase, *GPP* geranyl diphosphate, *LS* limonene synthase, *NPP* neryl diphosphate, *SOLPN* α-phellandrene synthase, *FDS* farnesyl diphosphate synthase. Similarly chemical structures of (−)-methanol, α-phellandrene; taxol, artemisinin and cucurbitacin C are shown as representative examples of terpenoids

Terpenoids are important for plant survival and also possess biological and pharmacological properties that are beneficial to humans. In plants, isopentenyl diphosphate (IPP) and dimethylallyl diphosphate (DMAPP) can be synthesized via two compartmentalized pathways. The mevalonic acid pathway of terpenoid biosynthesis operates in cytosol, the endoplasmic reticulum and peroxisomes (Carrie et al. 2007; Hemmerlin et al. 2003; Dudareva et al. 2006; Leivar et al. 2005; Merret et al. 2007; Sapir-Mir et al. 2008; Simkin et al. 2011; Lange and Ahkami 2013) (Fig. 1). The condensation of acetyl CoA three units leads to the synthesis of 3-hydroxy-3-methylglutaryl CoA, which later on produces mevalonic acid. The mevalonic acid converted to isopentenyl diphosphate through the process of the phosphorylation and decarboxylation. 3-hydroxy-3-methylglutaryl CoA reductase catalyzes the reduction of 3-hydroxy-3-methylglutaryl CoA to mevalonic acid (Luskey and Stevens 1985; Basson et al. 1988; Igual et al. 1992; Rodwell et al. 2000). In *Arabidopsis thaliana*, mevalonate-5-diphosphate is produced from mevalonic acid by the phosphorylation and the whole reaction is catalyzed by mevalonate kinase and phosphomevalonate kinase (Tsay and Robinson 1991; Lluch et al. 2000). Later on, the mevalonate-5-diphosphate decarboxylase catalyzes the conversion of mevalonate-5-diphosphate to isopentenyl diphosphate, which is the end product of mevalonic acid pathway of terpenoid biosynthesis (Dhe-Paganon et al. 1994) (Fig. 1).

Another part of terpenoid biosynthetic pathway starts in plastid by the condensation of pyruvic acid and glyceraldehydes-3-phosphate, which leads to the synthesis of 1-deoxy-d-xylulose 5-phosphate. The reaction is catalyzed by the enzyme as 1-deoxy-d-xylulose 5-phosphate synthase (Sprenger et al. 1997). The 1-deoxy-d-xylulose 5-phosphate reduced to 2-C-methyl-d-erythritol 4-phosphate by 1-deoxy-d-xylulose 5-phosphate reductoisomerase (Takahashi et al. 1998). The conjugation of 2-C-methyl-d-erythritol 4-phosphate and 4-cytidine 5-phosphate leads to the formation of 4-cytidine 5-phospho-2-C-methyl erythritol and the reaction catalyzed by the enzyme 2-C-methyl-d-erythritol 4-phosphate cytidyltransferase. The 4-cytidine 5-phospho-2C-methyl erythritol converted to 2-C-methyl erythritol 2,4-cyclodiphosphate by the enzyme 2-C-methyl erythritol 2,4-cyclodiphosphate synthase (Rohdich et al. 2000; Steinbacher et al. 2003; Herz et al. 2000; Calisto et al. 2007) (Fig. 1). All the enzymes of the 2C-methyl-d-erythritol-4-phosphate pathway are localized in plastids (Hseih et al. 2008; Surie et al. 2000). In the 1-deoxy-d-xylulose 5-phosphate pathway, the synthesis of hydroxymethylbutenyl 4-diphosphate took place from 2-C-methyl erythritol 2,4-cyclodiphosphate and the reaction was catalyzed by hydroxymethylbutenyl 4-diphosphate synthase. The hydroxymethylbutenyl 4-diphosphate directly converted into the isopentenyl diphosphate and dimethylallyl diphosphate mixture by the enzyme isopentenyl diphosphate and dimethylallyl diphosphate synthase (Baker et al. 1992; Cunningham et al. 2000).

In the steps of downstream process, mevalonate converted into IPP, which involves phosphorylations and decarboxylation events. The carried out reactions catalyzed by the following enzymes: mevalonate kinase, phosphomevalonate kinase and mevalonate diphosphate decarboxylase. Although the enzymes involved in these steps are thoroughly studied in yeast and various animal systems, very little information was reported in terms of their biochemical characterization in plants (Gershenzon and Kreish 1999). Recently, a *cis*-prenyl transferase, neryl phosphate synthase, was reported to provide precursor for monoterpene biosynthesis in several species of *Solanum* (Schilmiller et al. 2009; Lange and Ahkami 2013). A condensation of one molecule of DMAPP with two molecules of IPP generates farnesyl diphosphate (C_15_), the direct precursor of most sesquiterpenes, which is catalyzed by farnesyl diphosphate synthase. Plant genomes appear to encode various farnesyl diphosphate synthase isoforms that localize to the cytosol, plastids, mitochondria or peroxisomes (Cunillera et al. 1997; Thabet et al. 2011). In tomato, a *cis*-prenyl transferase, farnesyl diphosphate synthase is localized to plastids of the glandular trichomes, where it is involved in the biosynthesis of sesquiterpene volatiles (Salland et al. 2009). Diterpenes are formed from geranyl geranyl diphosphate which itself is synthesized by the catalysis of geranyl geranyl diphosphate synthase from DMAPP and three molecules of IPP. Isoforms of this enzyme have been reported to occur in plastids, the endoplasmic reticulum and mitochondria (Thabet et al. 2012; Sitthithaworn et al. 2001; Okada et al. 2000; Cheniclet et al. 1992). Terpene synthases often catalyze the formation of multiple products from a prenyl diphosphate substrate, resulting from a catalytic mechanism that involves highly reactive carbocation intermediates (Degenhardt et al. 2009). In general, monoterpene synthases are localized on plastids, whereas sesquiterpene synthases are found in the cytosol (Chen et al. 2011; Aharoni et al. 2003). A mitochondrial localization was determined for a terpene synthase in tomato (*Solanumlycopersicum*), but the in vivo substrate is currently unknown (Falara et al. 2011). The terpenoid skeletons are further functionized through redox, conjugation and other related reactions (Fig. 1).

**Fig. 2:**
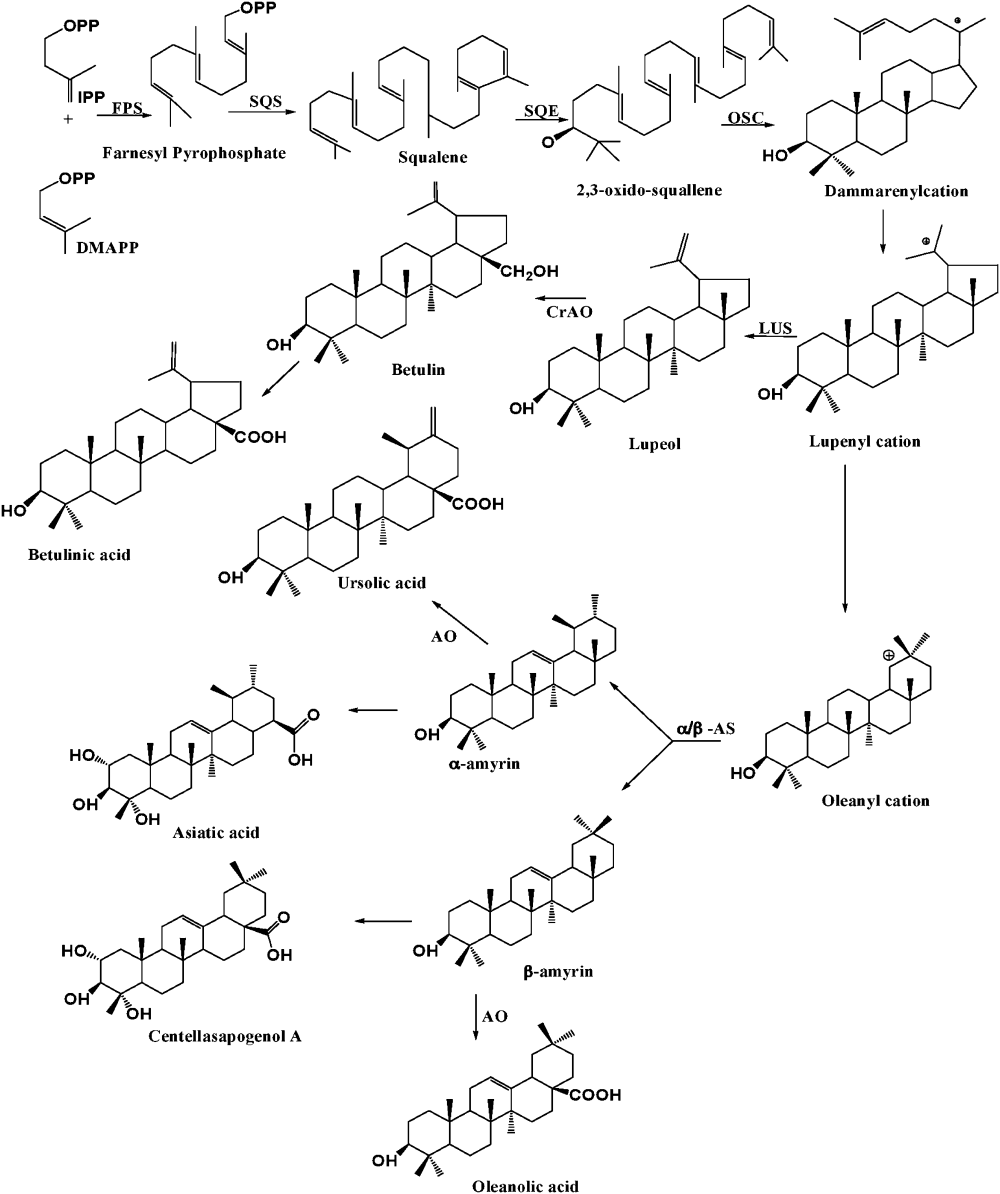
Schematic overview of triterpenoid biosynthesis. Farnesyl diphosphate synthase (FPS) isomerizes isopentenyl diphosphate and dimethylallyl diphosphate (DMAPP) to farnesyl diphosphate, while squalene synthase converts to squalene. Squalene epoxide oxidizes the squalene to 2,3-oxidosqualene. Oxidosqualene cyclase (OSC) catalyzes 2,3-oxidosqualene through cationic intermediates to one or more cyclic triterpene skeletons. The other enzymes involved in the biosynthesis include α/β amyrin synthase (α/β AS) which can also form the lupenyl cation but further ring expansion and rearrangements are required before the deprotonation to α/β amyrin, the precursors of sapogenins. α-Amyrin oxidase involved in biosynthesis of ursolic acid and oleanolic acid

The class of triterpenes includes sterols and triterpenoids, which can be synthesized as saponins and sapogenins sufficient amount in plants (Sparge et al. 2004). The linear triterpene squalene is derived from the reductive coupling of the two molecules of farnesyl pyrophosphate (FPP) by squalene synthase. Squalene is later on oxidized biosynthetically by the other enzyme squalene epoxidase to generate 2,3-oxidosqualene. 2,3-oxidosqualene converted to triterpene alcohols or aldehydes by oxidosqualene cyclases (Phillips et al. 2006; Jenner et al. 2005; Haralampidis et al. 2002). In plants, triterpenoid biosynthetic diversity has been developed and their diverse genomes encode multiple oxidosqualene cyclase enzymes to form these triterpene skeletons (Fig. 2). The level at which the structural diversity of triterpenes is generated, depends on the cyclization of 2,3-oxidosqualene by different oxidosqualene cyclases such as lupeol synthase (LS) and α/β-amyrin synthase (Mangus et al. 2006; Sawai and Saito 2011). All triterpene synthases appear to have diverged from cycloartenol synthase gene (Zhang et al. 2003), but an independent origin for β-AS in dicots and monocots has also been reported (Phillips et al. 2003; Moses et al. 2013).

The ursolic acid, oleanolic acid and betulinic acid are likely to be derived from α-amyrin, β-amyrin and lupeol, respectively, followed by successive oxidation (Augustin et al. 2011) at the C_28_ position. It has been shown that the triterpene skeletons (α-amyrin, β-amyrin and lupeol) are cyclized from 2,3-oxidosqualene, a common precursor of phytosterols and triterpenoids (Abe et al. 1993). Oxidosqualene cyclases yielded one specific product, such as lupeol synthase (Shibuya et al. 1999; Guhling et al. 2006; Moses et al. 2013), β-amyrin synthase (Kushiro et al. 1998; Kirby et al. 2008; Shibuya et al. 2009) and α-amyrin synthase (Muffler et al. 2011), cycloartenol synthase (Hayashi et al. 2000) and cucurbitadienol synthase (Shibuya 2004). Following the formation of the carbon skeletons, the triterpene alcohols are modified by various cytochrome P450s, dehydrogenases, reductases and other modification enzymes, while some triterpenoids are synthesized in all plant cells. Laticifers are elongated epithelial cells that produce chemically complex latex, which can consist of a polyterpenoids such as natural rubber (Beilen and Poirier 2007). Trichomes which are glandular in nature, are generally the known as storage organs of terpenoids and/ or phenolic compounds (Lange and Turner 2013).

The synthesis of isopentenyl diphosphate and dimethylallyl diphosphate, both are intermediates of terpenoid biosynthesis, is compartmentalized. The mevalonic acid pathway operates in cytosol, which is responsible for the formation of sesquiterpenes and sterols; 1-deoxy-d-xylulose 5-phosphate (DXP/MEP) pathway operates in the plastids, involved in the synthesis of monoterpenes, diterpenes and some sesquiterpenes as well as plastoquinones (Laule et al. 2003). In plants, mevalonic acid pathway’s enzyme localization is also fragmented. The 3-hydroxy-3-methylglutaryl CoA reductase and squalene synthase are localized in the endoplasmic reticulum (Leivar et al. 2005; Sapir-Mir et al. 2008; Busquets et al. 2008) while the acetoacetyl CoA is also important enzyme of mevalonic acid pathway, localized in peroxisomes (Reumann et al. 2007). In contrast, the DXP pathway enzymes are localized in plastids of cyanobacteria (Ginger et al. 2010). However, after constant observations of certain uncertainties, it has been widely accepted that initial reactions of DXP pathway are catalyzed in the cytosol while remaining reactions operated in the plastid. In red algae and the *Cyanophora paradoxa*, both mevalonic acid and DXP pathway run concurrently (Grauvogel and Peterson 2007). However, it has been proved that both the pathways are not separated spatially. The taxol (sesquiterpenes) is synthesized by the both mevalonic and DXP pathways (Adam and Zapp 1998; Wang et al. 2003). The unidirectional proton symport system of export of terpenoid intermediates and their involvement between cytosol and plastid pathway have been proved by the Ca^2+^ gated channel (Bick and Lange 2003). Laule et al. (2003) have suggested in their experiment that some limiting plastidial membrane transporters must be operated in the exchange of terpenoid intermediate’s exchange in between cytosol and plastid (Liao et al. 2006).

## Defense responses of terpenoids in plants

Plant kingdom has direct and indirect defense responses when they come in contact of microbial pathogens. The direct mode of defense mechanism includes physical structures like as trichomes, thorns as well as accumulation of phytochemicals that have antibiotic activities. The compounds such as phytoalexins are low-molecular-weight compounds that are produced as part of plant defense mechanisms. In few plant species the diterpenes and sesquiterpenes act as phytoalexins, e.g., 14 diterpene phytoalexins have been investigated from *Oryza sativa*. These phytoalexins can be grouped into four types—monilactones A and B (Hwang and Sung 1989), oryzalexins A–F (Peters 2006) and oryzalexin S (Tamongani and Mitani 1993). Polycyclic diterpenoids are synthesized from geranyl geranyl diphosphate via the intermediate hydrocarbon precursors (e.g., 9-β-pimara-7, 15-diene, stemar-13-ene, ent-sandaracopimaradiene and ent-cassa-12, 15-diene). All these natural products are accumulated in leaves in response to inoculation with the pathogenic blast fungus, *Magnaporthe grisea,* or ultraviolet irradiation and exhibit antimicrobial properties (Prisic et al. 2004).

Indirect mode of defenses indicates that the plants have characteristics to defend against herbivores indirectly by enhancing the effectiveness of natural enemies of the herbivores. One of the most amazing examples of the plant indirect defense is the release of the blend of specific volatiles, which attract the carnivores of herbivores, after herbivore attacking. More attention has been paid in case of corn, lima bean, poplar and cotton that are well studied with genetics, biochemical, physiological and ecological approaches (Rodriguez-Saona et al. 2003; Arimura et al. 2004a; Mithofer et al. 2005; Schnee et al. 2006). In an olfactometer assay, the transgenic *Arabidopsis* plants used as odor sources, females of the parasitoid *Cotesia marginiventris* learned to exploit the TPS10 sesquiterpenes to locate their lepidopteran host (Schnee et al. 2006). When a strawberry nerolidol synthase gene was expressed in *Arabidopsis* transgenic plants emitted two new terpenoids (3,S)-(*E*)-neridol and its derivative (*E*)-4,8-dimethyl-1,3-7-nonatriene [(*E*)-DMNT] and attracted more carnivorous predatory mites (Kappers et al. 2005). The capacity to produce deterrents to insects from plant-derived terpenoids is typical of some *Chrysolina* species. Because feeding of herbivores alters the aromatic profile of essential oil-producing plants like *Mentha aquatica*, the issue is both economically and ecologically relevant (Burse et al. 2009; Atsbaha Zebelo et al. 2011).

Airborne terpenoids are also critical components of plant defense responses to abiotic and biotic stresses (Unsicker et al. 2009; Vickers et al. 2009). From agronomic perspective, crop losses due to insect infestation are a significant issue (El-Wakeil et al. 2010). Insecticide applications are most common and effective strategy for control of insects, but some of the agrochemicals have undesirable side effects on useful insects and can pose long-term risks to the environment (Dedryver et al. 2010; Zulak and Bohlmann 2010).

When patchoulol synthase (PTS), a sesquiterpene synthase from *Pogostemon cabli* L., was targeted to the cytosol in transgenic tobacco (*Nicotiana tabacum* L. cv. Xanthi), only small amounts of the expected product, patchoulol, were detected. When the same gene was expressed coordinately with an additional copy of farnesyl diphosphate synthase, the patchoulol accumulation in transgenic tobacco also remained very low (Wu et al. 2006), while both gene products patchoulol synthase and farnesyl diphosphate synthase were targeted to plastids, a patchoulol accumulation was increased, which appeared to be volatilized. It was also observed that volatile emitted from these transgenic plants significantly deterred tobacco hornworms and pine beetles from feeding on leaves (Wu et al. 2006; Bohlmann 2012).

## Plant terpene synthases

The investigations of terpene synthases have been an interesting and active area of plant metabolic engineering research and may genes have been isolated from various plant species (gymnosperms and angiosperms), including *Picea abies* (Martin et al. 2004), *Taxus media* (Wildung and Croteau 1996), *Arabidopsis thaliana* (Chen et al. 2003; Degenhardt et al. 2009), *Cucumis sativus* (Mercke et al. 2004), and *Nicotiana attenuata* (Facchini and Chappel 1992). The total numbers of terpene synthases reported from *Thapsia laciniata* are 8 monoterpene and 5 sesquiterpene (Drew et al. 2013) which are slightly larger than the number reported in *Arabidopsis thaliana* and *Artemisia annua* (Tholl and Lee 2011). Several sesquiterpene synthases have also been cloned and characterized from maize (Kölner et al. 2004). In general, the lengths of monoterpene synthases are between 600 and 650 amino acid residues and are 50–70 amino acids are larger than sesquiterpene synthases (Martin et al. 2004). Most diterpene synthases are approximately 210 amino acids longer than monoterpene synthases because of an additional internal element that is conserved in both sequence and position (Prisic et al. 2004). All the terpene synthases contain the aspartate-rich DDxxD motif involved in the coordination of divalent metal ions for substrate binding (Lesburg et al. 1997). The terpene synthases are further sub-classified into four sub-families—TPSa, TPSb, TPSd and TPSg. The TPSa family consists of angiosperm terpene synthases (Bohlmann et al. 1998; Dudareva et al. 2003), the TPSb family contains angiosperm monoterpene synthases (Bohlmann et al. 1998; Dudareva et al. 2003), the TPSb includes angiosperm monoterpene synthases (Bohlmann et al. 1998; Dudareva et al. 2003), TPSd of gymnosperm monoterpene synthases (Bohlmann et al. 1998; Dudareva et al. 2003) and TPSg *Antirrhinum majus* monoterpene synthases (Bohlmann et al. 1998; Chen et al. 2003) also share common evolutionary origin.

The triterpene synthases lead to synthesis of tricyclic, tetracyclic and pentacyclic molecules in complex of concerted reaction steps catalyzed by single enzyme. The sterol and triterpenoid biosynthetic pathways diverged at some point, depending on the involvement of the type of the oxidosqualene synthases. Cyclization of 2,3-oxidosqualene in the chair–boat–chair conformation leads to protosteryl cation intermediate, sterol precursor, via the synthesis of cycloartenol or lanosterol in plants (Kolesnikova et al. 2006; Suzuki et al. 2006), while in contrast to 2,3-oxidosqualene in the chair–chair–chair conformation is cyclized into a dammarenyl carbocation intermediate, which subsequently gives rise to diverse triterpenoid skeletons after further re-arrangements. Many different types of oxidosqualene synthases have been isolated from various plant species including lanosterol synthase (Baker et al. 1995; Sung et al. 1995), cycloartenol synthase (Bach 1995; Kawano et al. 2002), lupeol synthase (Hayashi et al. 2004; Segura et al. 2000) and β-amyrin synthase (Hayashi et al. 2001; Iturbe-Ormaetxe et al. 2003). Besides these synthases, some multifunctional triterpene synthase have also been characterized from other different plant species (Basyuni et al. 2006; Shibuya et al. 2007).

## Phylogenetic analysis of terpenoid synthases

On the basis of the phylogeny, the gymnosperm terpene synthases have been subdivided into three distinct clades—TPS-d1 to TPS-d3. The TPS-d1 subclade are (−)-α/β-pinene synthases, (−)-linalool synthases and (*E*)-α-farnesene synthases; in TPS-d2 clade are longifoline synthase and in TPS-d3 clade are levopimaradiene/abietadiene synthases and isopimaradiene synthase (Martin et al. 2004). The functional identification of spruce terpene synthase genes account for several terpenoid compounds of the oleoresin and volatile emissions. Many terpene synthase genes (*TPSd*) of terpenoid metabolism, especially *ent*-copalyl diphosphate synthase and *ent*-kaurene synthase gene appear to be expressed as single copy genes (Bohlmann et al. 1999). These primary metabolism terpene synthase genes are basal to the specialized metabolism genes and are the descendants of an ancestral plant diterpene synthase similar to the non-vascular plant as *Physcomitrellapatens* (Hayashi et al. 2006; Keeling et al. 2010, 2011).

Sesquiterpene synthases sequence of roots of*Cycus* species was investigated and it was found that gymnosperms form a distinct group from the angiosperms, which displayed a pattern that seemed to be influenced by the types of products of different plant species. By the phylogenetic analysis, it can be predicted that α-copaene synthase gene is more homologous to germacrene B, germacrene D and valencene synthase gene (Hiltpold and Turlings 2008; Wen et al. 2012). A sesquiterpene synthase gene that produces α-copaene as its sole reaction product has been reported. This enzyme is highly expressed in potato and correspondence to the difference in tuber flavor between two cultivars of potato (Ducreux et al. 2008; Zapta and Fine 2013).

The terpenoid synthases of primary metabolism, (−)-CDP synthase (Sun and Kamiya 1994; Ait-Ali et al. 1994; Bensen et al. 1995) and kaurene synthase B (Yamaguchi et al. 1996), are only distantly related to those of secondary metabolism, including members of sub-families TPSa, TPSb and TPSd. However, all plant terpene synthases share a common evolutionary origin and it appears that the bifurcation of terpenoid synthases of primary and secondary metabolism occurred before separation of angiosperms and gymnosperms. Terpene synthases of secondary metabolism constitute the most extensively studied TPS sub-families including TPSa, TPSb, TPSd and the distant and possibly ancient TPSf branch containing linalool synthase (Bohlmann et al. 1998; Chen et al. 1996). Valencene synthase (Sheron-Asa et al. 2003) and 5-epi aristolochene synthase (Back and Chappel 1996) are related to one another based on the biosynthesis of sesquiterpenes within the eremophilene class of compounds. The *Magnolia* possesses a single intron positioned near the 5′ region of the gene, similar to the first intron in all other three classes of terpene synthases from plants. The intron found in fungal trichodiene synthase gene is inserted into the middle of the trichodiene synthase gene and is not spatially oriented similar to the insertion site of the first intron in any of the plant genes including *Mg25* (Trapp and Croteau 2001; Lee and Chappel 2008). The dendrogram analysis was conducted to determine the evolutionary relatedness of chamomile terpene synthases to those of others Asteraceae. MrTPS1, MrTPS2, MrTPS3 and MrTPS5 were found to belong to the TPSa sub-family covering angiosperms, whereas MrTPS4 fell into the TPSb sub-family covering angiosperm monoterpene synthases (Irmisch et al. 2012). The monoterpenes are formed in plastids and the nucleus-encoded monoterpene synthases are targeted by N-terminal transit peptides of approximately 40–70 amino acids which reside upstream of the conserved RRx8W motif and are cleaved during import from the nucleus. (Williams et al. 1998; Turner et al. 1999). In contrast to the multiple closely related AtTPS of the TPSa and TPSb groups, only one AtTPS member is found in each of three sub-families TPSc, TPSe and TPSf (*Arabidopsis thaliana* terpene synthases). The copalyl diphosphate synthases show between 45 and 55 % identity. The AtTPS GA2 enzyme (Yamaguchi et al. 1998) is a diterpene synthase of the TPSe sub-family of kaurene synthases. Finally AtTPS04 has a TPSf type primary structure reminiscent of that of linalool synthase from *Clarkia breweri* (Dudareva et al. 1996). It has been suggested that AtTPS04 is an orthologue of this linalool synthase in *Arabidopsis thaliana* (Cseke et al. 1998; Aubourg et al. 2002). The comparison of Grtps (grape fruit terpene synthases) amino acid sequence with the sequences of other terpene synthase indicated that this *Grtps* cDNA is truncated at the 5′ terminus and that the truncation represents 18 amino acid residues of the presumptive transit peptide region at the N-terminus of the deduced protein. The peptide is found in both monoterpene and diterpene synthases and supposedly facilitates the import of these nuclear encoded gene products into plastids, a process that involves cleavage of the preproteins to the mature active enzymes (Mau and West 1994; Vogel et al. 1996; Jia et al. 2005). The phylogenetic analysis of large number of *Vitis vinifera* terpene synthase genes resolved a bifurcation of TPSb and TPSg sub-families at a juncture that was previously ambiguous and had misclassified some TPSg genes as TPSb members. Later on it was concluded that grapevine geraniol and linalool synthase matches with basil geraniol and linalool synthase and showed same proximity which indicates that these TPS functions already existed have evolved from same ancestor (Martin et al. 2010).

*Azadirachta indica* and *Citrus* belong to the order Rutales and the phylogenetic studies reaffirmed their taxonomic closeness. Additionally, phylogenetic studies grouped *A. indica* with *Melia* species, one that is also known to harbor bioactive compounds suggesting a common evolutionary process with regard to synthesis of these compounds in Meliaceae. The repeat analysis showed low repeat content in *A. indica* genome compared with other sequenced angiosperms. This could have been due to presence of xenobiotic terpenoids specific to the plant, which might have been a major impediment for horizontal gene transfer (Richardson and Palmer 2007; Krishnan et al. 2012). The phylogenetic analysis of cycloartenol synthase, lupeol synthases and the dicot β-amyrin synthase reported them as multifunctional enzymes. These enzymes have same specificity clusters by which authors have suggested a molecular evolution mechanism for lupeol synthase and β-amyrin synthase arising from a common ancestral cycloartenol synthase (Shibuya et al. 1999; Zhang et al. 2003).

The increasing diversification of the cyclization reaction sequence from the dammarenyl to the oleanyl cation via the lupenyl cation is consistent with this evolutionary scheme. MdOSC1, MdOSC2 and MdOSC3 are located with in the group of enzymes that produce a dammarenyl cation intermediate in *Malus domestica*. In MdOSC1 and MdOSC3 cluster, lupeol synthases are more related to β-amyrin synthases than to lupeol synthases (Basyuni et al. 2007; Guhling et al. 2006; Brendolise et al. 2011). This new class of lupeol synthase includes BgLUS, RcLUS and multifunctional triterpene synthase KcMS and another putative OSC (EtOSC—*Euphorbia terucalli* triterpene synthase) for which no triterpene synthase activity has been detected when it is expressed in yeast (Kajikawa et al. 2005).

The phylogenetic analysis using neighbor-joining methods showed that SlTTS1 and SlTTS2 (*Solanum lycopersicum*) terpene synthases are more closely related to each other than to any other oxidosqualene cyclases and they together with the *Panax ginseng* β-amyrin synthases (Kushiro et al. 1998) form the subclad within a group of oxidosqualene cyclase enzyme that were all characterized as β-amyrin synthases from different plant species. The intron patterns and exon lengths of the two *S1TTS1* genes are very similar to those of the other oxidosqualene cyclases, while *S1TTS2* gene organization most closely resembles OSC3 of *Lotus japonicus* (Sawai et al. 2006; Wang et al. 2011). The close match in localization of transcripts and metabolites makes it very likely that *S1TTS1* and *S1TTS2* genes dedicated entirely making the triterpenoids destined for the cuticular wax of the fruit surface. This major biological function can be assigned to the OSCs; the cuticular triterpenoids contribute significantly to the chemical composition and to the ecophysiological properties of the fruit cuticle (Vogg et al. 2004; Isaacson et al. 2009). It should be noted that similar biological functions had previously been attributed to a few other OSCs, for example, a glutinol synthase and a friedelin synthase from *Kalanchoedaigremontiana* (Wang et al. 2010).

It has been reported that 13 *Arabidopsis thalianaOSC* genes and the 11 triterpene synthase genes are grouped into one functional group. Furthermore, 20 out of 36 Poaceae *OSC* genes were also assigned either to the pentacyclic triterpene synthase-like group based on the characterized β-amyrin synthase from *Avena* species (Haralampidis et al. 2001; Qi et al. 2004) or to the rice isoarborinol synthase group (Xue et al. 2012). In *Arabidopsis thaliana*, a tandem cluster on chromosome 1 containing four homologous *OSC* genes, *At1g78950*, *At1g78955/CAMS1*, *At1g78960/LUP2* and *At1g78970/LUP1*, is likely to have arisen by three tandem duplication events. Another tandem duplicate gene pair *At4g15340* and *At4g15370*, encoding arabidiol synthase and baruol synthase, respectively (Xiang et al. 2006; Lodeiro et al. 2007), is located on *A. thaliana* chromosome 4. Indeed, most triterpene synthase genes in the Poaceae family appear to have arisen from *CS* genes by the D3 gene duplication event, which caused the divergence of the 20 triterpene synthase genes (D3-2) from 12 *CS* genes and other closely related genes form group D3-1. The D3 duplication event is highly likely to have been a tendem duplication that occurred during the ancient Poaceae genome before the ρ whole genome duplication, which was estimated to have occurred between 117 and 50 mya (Gaut 2002; Yu et al. 2005; Lescot et al. 2008; Jaio et al. 2011).

In pairwise comparison of all predicted *Citrus* terpene synthases with all *Arabidopsis* AtTPS proteins, it was found that the overall sequence identity varies widely from 18 to 91 %. *Citrus* terpene synthase EST contigs were long enough to allow the complete encoded protein sequences to be deduced (ranged from 547 to 617 amino acids), which corresponds to the size of known monoterpene synthases, sesquiterpene synthases and diterpene synthases of secondary metabolism (Bohlmann et al. 1998; Aubourg et al. 2002). Most terpene synthases encoded by class-III genes contain variations of a conserved motif RR(x)8W, close to the N-terminus (Dornelas and Mazzafera 2007).

2-Methyl-3-buten-2-ol (MBO-hemiterpene) is a five-carbon alcohol produced and emitted by plant species of pine in large quantities. The gene most closely related to MBO synthase is a linalool synthase from *Piceaabies* with which MBO shares 82 % amino acid identity. Also closely related to MBO synthase are farnesene synthase from *P. abies* and *P. taeda*. These enzymes form a strongly supported clade of related enzymes producing MBO, linalool and *E*-α-farnesene nested within what is otherwise a clade dominated by enzymes producing cyclic monoterpene (Gray et al. 2011). MBO synthase and isoprene synthase comparison clearly demonstrate that hemiterpene synthase evolved independently in gymnosperms and angiosperms. The MBO synthase clusters with gymnosperm monoterpene synthase, isoprene clusters with angiosperm monoterpene synthases and these gene families diverged between 250–290 million years ago (Martin et al. 2004).

## Regulation of terpenoid biosynthesis

The role of light and temperature in modulating a range of terpenoids and the corresponding transcripts has been reported, but there is no universal behaviour and it varies depending upon the type of metabolites as well as plant species. The 3-hydroxy-3-methylglutaryl CoA reductase is stimulated by light in *Triticumaestivum* (Aoyogi et al. 1993), pea (Wong et al. 1982) and potato (Korth et al. 2000), but down-regulated by light in *Lithospermumerythrorhizon* (Lange et al. 1998). The effects of light as promoter on 3-hydroxy-3-methylglutaryl CoA reductase activity has been documented and also the light-mediated alteration in 3-hydroxy-3-methylglutaryl CoA reductase transcripts (Learned and Connolly 1997; Kawoosa et al. 2010). Rodrìguez-Concepción (2006) suggested about the light-dependent regulation of terpenoid biosynthesis during the early stages of development in *Arabidopsis thaliana*. As per their model, the seedlings which were grown in the dark obtain lower level of precursors for the synthesis of sterols from the mevalonic acid pathway. Some of the prenyl diphosphates of the mevalonic acid pathway might be translocated to the plastid for the synthesis of carotenoids and gibbrellins; while those seedlings were grown in the light, the activity of mevalonic acid pathway increased and the isoprenoid precursors are not required by the plastid (Vranova et al. 2012). In *Artemisia annua*, the discharging of β-pinene fluctuates as per the rhythm of day as well as night and it is higher in the day light than night (Lu et al. 2002). The all terpenoid compounds of *Arabidopsis thaliana* flowers showed clear diurnal emission patterns (Aharoni et al. 2003).

Jasmonate was reported to be general inducer of biosynthesis of plant secondary metabolite (Memelink et al. 2001; van der Fits and Memelink 2000). Jasmonate and its derivative methyl jasmonate were shown to induce the terpenoid indole biosynthesis in suspension cell cultured with auxin and to enhance the terpenoid indole alkaloid production when cells were cultured in an auxin-free medium (Gantet et al. 1998). Genes involved in secologanin biosynthesis (*Crdxs*, *Crcpr*) were upregulated by methyl jasmonate as well as most of the known other genes of terpenoid indole alkaloid biosynthesis pathway (Hedhili et al. 2007; Siamaru et al. 2007; Zhao et al. 2004). Downstream of the conserved jasmonate hormone perception and initial signaling cascade, species-specific transcriptional machineries exist that regulate the transcriptional activity of specific biosynthetic genes (Pauwels et al. 2009; Pauwels and Goosens 2011). A few transcriptional factors regulated by the jasmonate hormone signaling cascade that activate the transcription of sesquiterpenoid biosynthetic genes have already been reported (De-Geyter et al. 2012). The triterpenoid contents in *Ocimum basilicum* was produced higher in quantity after the treatment with methyl jasmonate rather than control plants. The exogenous treatment of methyl jasmonate affected the production of terpenoids by regulating the terpene synthase genes (Li et al. 2007; Prins et al. 2010).

It is well known that the monoterpene synthase gene *SIMTS1* activity is induced by jasmonic acid (van Schie et al. 2007). The jasmonic acid has been shown to increase trichome density on newly formed leaves of *Arabidopsis* and tomato (Boughton et al. 2005; Traw and Bergelson 2003). The production of acyl sugars on the leaf surface of *Daturawrightii* plants increased without affecting trichome density (Hare and Walling 2006). The expression levels of most of the genes of the mevalonic acid pathway and monoterpene or sesquiterpene synthesis follow the same profile during development, suggesting coordinated regulation of terpenoid biosynthesis at the gene level. However, although expression of these genes is relatively trichome specific, the expression profiles do not alter metabolic accumulation during development, suggesting that terpenoid synthesis is not regulated at the transcript level in tomato trichomes but rather involves other (post-transcriptional) regulatory mechanisms. Similarly, only a loose correlation between terpenoid pathway gene expression and enzyme activity has been found in *Ocimumbasilicum* terpenoid metabolism (Iijima et al. 2004; Besser et al. 2008). Jasmonic acid is essential for induction of defenses in glandular trichomes. Production of many trichome metabolites is also regulated tightly by transcriptional control, thereby allowing for temporally regulated emission of plant volatiles (Dudareva et al. 2006; Glas et al. 2012).

Methyl jasmonate is a cyclopentanone ring bearing lipophilic hormone synthesized in plants from octadecanoid pathway and they play role in development of responses to biotic stress (Creelman and Mullet 1997). The changes in accumulation of terpene synthase transcripts were also observed in methyl jasmonate-treated Norway spruce; this supports the view that the transcription of terpene synthase genes also regulated by this hormone (Fäldt et al. 2003). The accumulation of taxoid (diterpene taxadiene) was enhanced by supplementation of methyl jasmonate in cell cultures of *Taxus* (Ketchum et al. 1999; Phillips et al. 2006). Traumatic ducts are specialized anatomical structures for the accumulation of resin terpenes, which are formed in Norway spruce and other conifer plants. Like other resin ducts, traumatic ducts are lined with epithelial cells thought to be the site of terpene biosynthesis. The development of traumatic ducts in xylem of Norawy spruce, induced by the treatment of methyl jasmonate was similar in fashion, which caused by the attack of fungal elicitation and mechanical wounding (Krokene et al. 2008; Herrera et al. 2005; Martin et al. 2002). The morphological changes are accompanied by an increase in monoterpene and diterpene synthase activity peaking at the highest rate at 10–15 days after treatment of methyl jasmonate (Martin et al. 2002). The significant increase in the resin terpenoid quantity in bark and wood of Norway spruce followed by methyl jasmonate treatment has also been reported (Martin et al. 2004; Miller et al. 2005).

The level of mRNAs of squalene synthase and β-amyrin synthase was upregulated by adding methyl jasmonate to *Glycyrrhiza glabra* cell cultures and it was observed that the level was higher 3 days after the treatment and lasted for 7 days. The mRNA levels of cycloartenol synthase and oxidosqualene cyclase, which are involved in the biosynthesis of phytosterols, were relatively constant (Hayashi et al. 2003). *Artemisia annua* plants treated with methyl jasmonate showed only slight change in the transcription levels of the control plants. 3-hydroxy-3-methylglutaryl CoA reductase gene expression decreased 1.5-fold at 24 h and then increased threefold by 48 h (Mehjerdi et al. 2013).

When the zeatin and ethylene were added together to the culture medium of *Catharanthus roseus* cell cultures, the mRNA level of mevalonic acid pathway genes coordinately increased in suspension culture cells (Papon et al. 2005). In addition, the zeatin stimulates the bioconversion of exogenic secologanin to the terpenoid indole alkaloid ajmalicine, suggesting that cytokinin may also act on other downstream enzymatic steps of the terpenoid indole alkaloid biosynthesis pathway (Decendit et al. 1992). Similarly, the ethylene treatment itself induces the formation of traumatic ducts in *Pseudotsugamenziesii* and *Sequoiadendrongiganteum* (Hudgins and Franceschi 2004) and stimulates the accumulation of β-thujaplicin in *C*. *lusitanica* at low levels (Phillips et al. 2006).

Dudareva et al. (2003) reported that the biosynthesis and emission of the monoterpenes (*E*)-β-ocimene and myrcene in *Antirrhinum majus* flowers correlate with specific expression patterns of the lobes of flower petals during floral development, with the highest transcripts levels detected at day four post anthesis. In *Arabidopsis* flowers, monoterpene and sesquiterpene synthases are not expressed in flower petals; instead their expression is limited to the stigma, anthers and sepals (Tholl et al. 2005). Many monoterpene and sesquiterpene synthase genes have been reported from terpene accumulating cells and tissues such as leaf glandular trichomes of *Citrus* and grapes (Picaud et al. 2005; Lücker et al. 2004; Shimada et al. 2004; Kai et al. 2006). Most of the terpene synthase genes belonging to TPSa and TPSb sub-families reached the highest expression in accordance with the peak of accumulation of the respective compounds, while in TPSg sub-family, only one gene for linalool synthase showed major transcript in the ripening of berries. The geraniol synthase had a peak of expression that started to increase and overcome the linalool concentration (Matarese et al. 2013; Chen et al. 2011; Falara et al. 2011).

**Fig. 3:**
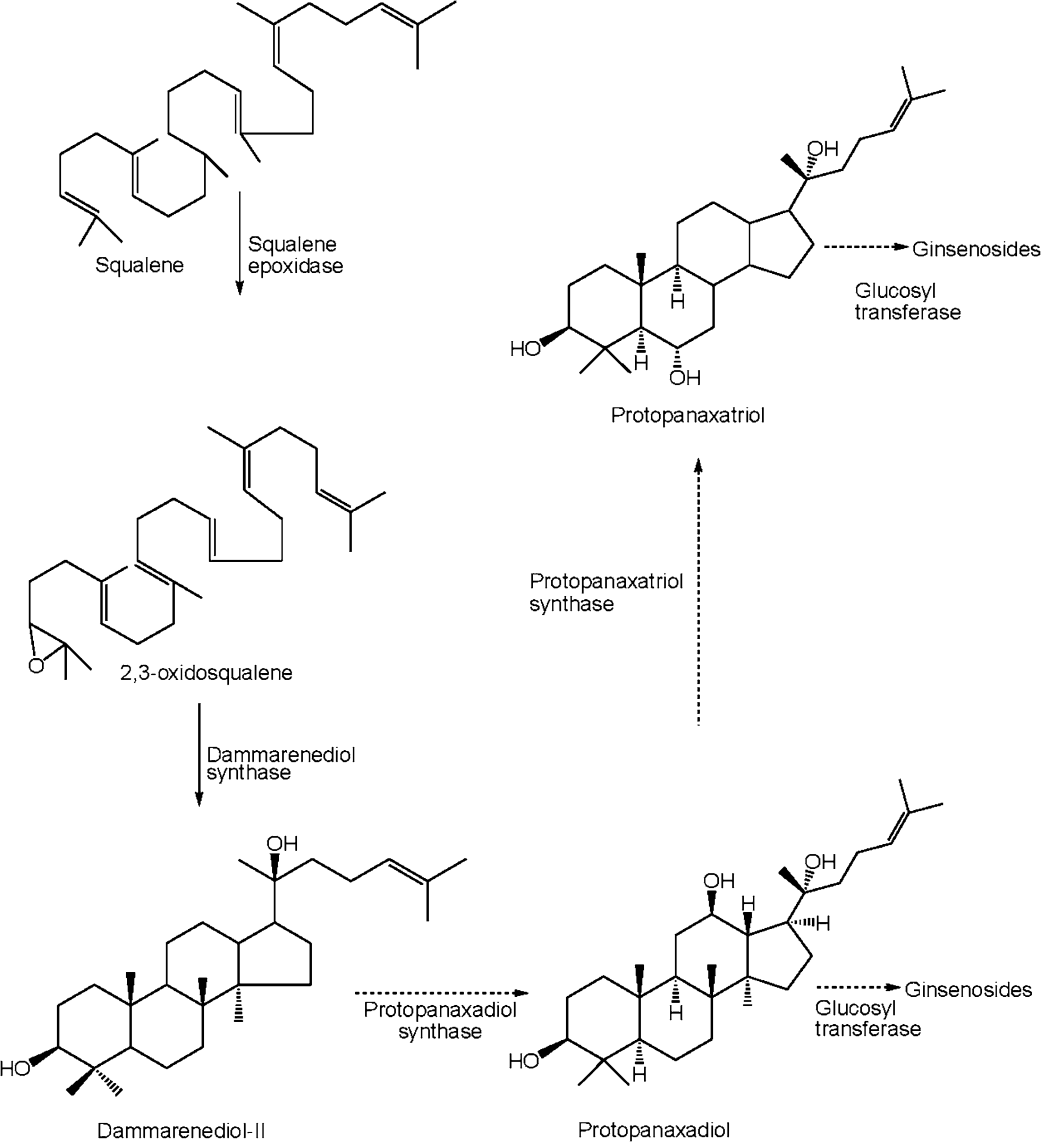
Biosynthetic pathways of ginsenosides from squalene in *P. ginseng*

**Fig. 4:**
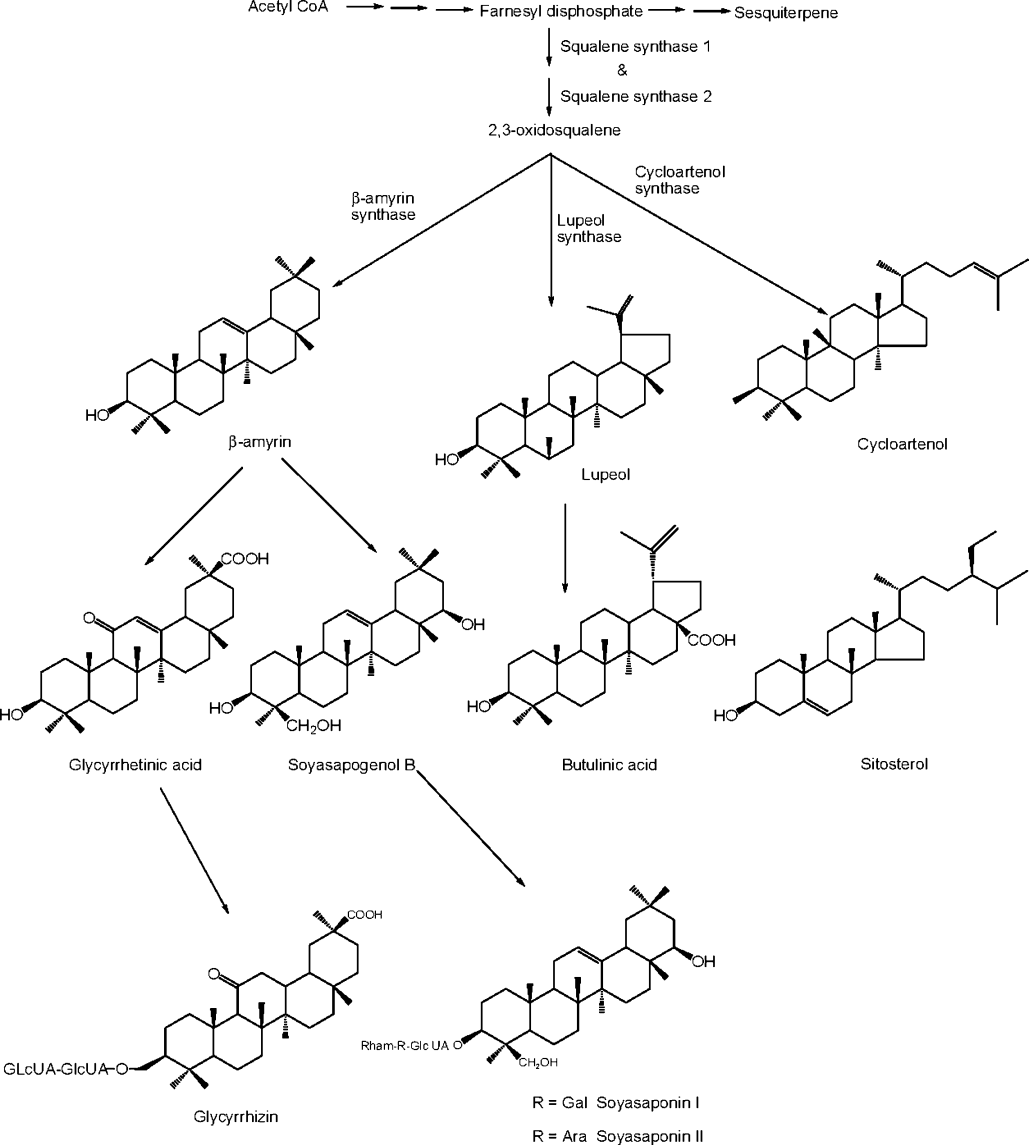
Biosynthetic pathways of glycyrrhizin in *Glycyrrhiza glabra*

Ginsenoside backbones are synthesized via the isoprenoid pathways where squalene acts as precursor. The squalene is synthesized by a series of several reactions with geranyl diphosphate synthase, farnesyl pyrophosphate synthase and squalene synthase through mevalonate pathway (Kuzuyama 2002) and subsequent reactions with squalene epoxidase yielded 2,3-oxidosqualene (Fig. 3). The cyclization of 2,3-oxidosqualene into dammarenediol and β-amyrin is catalyzed by oxidosqualene cyclases including dammarenediol-II synthase and β-amyrin synthase (Han et al. 2006, 2010; Tansakul et al. 2006; Kim et al. 2010). Both glycyrrhizin and soyasaponins share a common biosynthetic intermediate, β-amyrin (Fig. 3), which is synthesized by β-amyrin synthase, an oxidosqualene cyclase (OSCs). OSCs catalyze the cyclization of 2,3-oxidosqualene, a common intermediate of both triterpene and phytosterol biosynthesis (Abe et al. 1993; Haralampidis et al. 2002). In *Glycyrrhizaglabra* three OSCs: β-amyrin synthase, lupeol synthase and cycloartenol synthase are situated at the branching step for biosynthesis of oleanane-type triterpene saponins, lupane-type triterpene (betulinic acid) and phytosterol, respectively (Fig. 4). cDNAs of β-amyrin synthase (Hayashi et al. 2001) and cycloartenol synthase (Hayashi et al. 2000) have already been isolated from cultured cells of licorice (Kölner et al. 2004; Hayashi et al. 2003).

Terpenoid biosynthesis occurs within specific tissues or at specific stages of development in plants (Nagegowda 2010; Vranova et al. 2012). In many plant species those have glandular trichomes, specialized structures for secreted terpenoid natural products (Lange and Turner 2013). The glycyrrhizin accumulates only in underground organs, stolons and roots of licorice plants (Seki et al. 2008). Avenacins, the bioactive saponins in *Avenasativa* accumulate only in epidermis of roots, where they develop resistance to plant pathogenic fungal organisms (Haralampidis et al. 2001). The biosynthesis of avenacin genes are co-regulated and exclusively expressed in the epidermis of roots in which the avenacins are accumulated (Qi et al. 2006; Field and Osbourn 2008).

Sometimes the regulation of terpenoid biosynthesis is induced by herbivore feeding, attack by pathogen or abiotic stresses (Nagegowda 2010; Vranova et al. 2012). The enhancement of concentration of terpenoids in response to various abiotic stresses is often is mediated by an increase in transcriptional activity of the specific terpenoid biosynthetic genes (Tholl 2006; Nagegowda et al. 2004, 2010; Xi et al. 2012). This type of transcriptional response is controlled by complex signaling cascade in which jasmonate hormone play important role. The pathogen attack causes transcriptional and metabolic changes in plant cell cultures of *Medicagosativa* (Suzuki et al. 2005). The defence mechanism-related synthesis of terpenoids has been studied by various authors (Van Poecke et al. 2001; Rodriguez-Saona et al. 2003). The synthesis of several terpenoids in poplar is induced and emitted from the *Malacosoma disstria* infested leaves (Arimura et al. 2004a). *Tetranychus urticae* infests on the *Lotus japonicas* and after this it induces the emission of the (*E*)-β-ocimene and also accumulation of (*E*)-β-ocimene synthase gene transcript (Arimura et al. 2004b).

In addition to the transcriptional, developmental and spatiotemporal modulation of terpenoid biosynthetic genes, the post-translational regulation mechanisms were also reported in the biosynthesis of terpenoids. The HMGR activity, the specific enzyme that catalyzes crucial regulatory steps of the mevalonic acid pathway, is controlled at the protein level through the activity of protein phosphatase A or by the E3 ubiquitin ligase *SUD1* (Leivar et al. 2011; Doblas et al. 2013). The role of jasmonic acid in the induction of trichome-specific terpene synthases has been well reported (van Schie et al. 2007). Upon perception of pathogens or herbivores, signal transduction pathways are activated, which lead to induced defense responses.

## Terpenoids under clinical trials

Terpenes are the largest group of natural bioactive compounds including monoterpenes, sesquiterpenes, diterpenes, hemiterpenes and triterpenes. Out of these natural compounds, several terpenes are under studies of clinical trials, which are as follows:

### d-Limonene

Monoterpenes such as d-limonene and peryl alcohol prevent mammary, live and other types of cancers. The monoterpenes have several cellular and molecular activities that could potentially underlie their positive therapeutic index. The monoterpenes inhibit the isoprenylation of small G proteins. Such inhibitions could alter signal transduction and result in altered gene expression. When mammary cancers were initiated in rats by either the direct acting carcinogen *N*-methyl-*N*-nitrosourea or indirectly acting carcinogen DMBA, they could be prevented from developing if the carcinogen-exposed rats were fed d-limonene (Elson et al. 1988; Yoon et al. 2010).

d-Limonene is considered to have fairy low toxicity. It has been tested for carcinogenicity in mice and rats. Although initial results showed d-limonene increased the incidence of renal tubular tumours in male rats, female rats and mice in both genders showed no evidence of any tumor. Subsequent studies have determined how these tumors occur and established that d-limonene doses pose mutagenic or nephrotoxic risks to humans as well as human prostate cancers. In humans, d-limonene has demonstrated toxicity after single and repeated dosing for up to 1 year. Being a solvent of cholesterol d-limonene has been used to clinically to dissolve cholesterol containing gallstones (Igimi et al. 1991; Rabi and Bishayee 2009a, b). Because of its gastric acid neutralizing effect and its support of normal peristalsis, it has been also used for relief of heartburn and gastroesophageal reflux (Kodama et al. 1976; Sun 2007). In phase I clinical trial of orally administered d-limonene, 17 women and 15 men aged 35 to 78 with advanced metastatic solid tumors received an average of three treatment cycles of 21 days at dose ranging from 0.5 to 12.0 g/m^2^ body surface area. d-Limonene was slowly absorbed, the maximal plasma concentration being attained at 1–6 h. The mean peak plasma concentrations of d-limonene were 11–20 μmol/L and the predominant metabolites were perillic acid (21–71 μmol/L), dihydroperillic acid (17–28 μmol/L) and isomers of perillic acid. After reaching these peaks, the plasma concentration decreased according to first-order kinetics (Vigushin et al. 1998; Saldanha and Tollefsbol 2012). Carcinomas regress when d-limonene is added to the diet either when the tumour is small or still capable of spontaneously regressing. d-Limonene appears to act in cytostatic fashion. It is predicted that d-limonene inhibits the isoprenylation of small G proteins (Hogg et al. 1992; Gould et al. 1994; Miller et al. 2011).

### 1,8-Cineole

In humans, 1,8-cineole inhibits sensory irritations caused by octanol and methanol with sensitive volunteers. Both methanol and octanol are well-known chemicals causing skin irritation. The result that 1,8-cineole, whose ability to activate TRMP8 is lower than methanol, inhibited methanol-evoked skin irritation clearly suggests that inhibitory effects of 1,8-cineole are probably due to inhibition of TRPA1, but not activation of TRMP8 (Takashi et al. 2012; Bastos et al. 2011). 1,8-cineole not only reduces exacerbation rate but also provides clinical benefits as manifested by improved airflow obstruction, reduced severity of dyspnea and improvement of health status (Juergens et al. 1998, 2003). Therefore, it can provide a useful treatment option for symptomatic patients with COPD in addition to treatment according to the guidelines. The results have to be seen in context with socio-economic aspects. As COPD is an extremely costly disease and cause of major financial and social burden concomitant therapy with 1,8-cineole can be recommended. These finding correspond to the interpretation of the efficacy study with carbocysteine but not with acetylcysteine because this medication did not show a significant reduction of exacerbations (Zhang et al. 2008; Decramer et al. 2005; Worth et al. 2009).

### Boswellic acid

More recently extracts of resin enriched in pentacyclic triterpenoid known as boswellic acid have been employed as anti-inflammatory drugs (Anthoni et al. 2006). Pilot clinical studies do indeed suggest that boswellic acid promotes pain control and dampens inflammation in osteoarthritis and colitis and helps to control the brain oedema associated with radiotherapy of cerebral tumours; anti-inflammatory effects of rodent models have also been demonstrated (Gupta et al. 2001; Kimmatkar et al. 2003). Initial attempts to clarify the molecular target of boswellic acid in inflammatory disorders determined that keto-boswellic acid can inhibit 5-lipoxygenase in low molecular concentrations (Bhushan et al. 2007). This suggested that boswellic acid preparation might dampen inflammation by blocking leukotriene synthesis (Joos et al. 2006). In vitro studies reveals boswellic acid in a dose-dependent manner blocks the synthesis of pro-inflammatory 5-lipoxygenase product including 5-hydroxyeicosatetraenoic acid and leukotriene B4, which cause bronchoconstriction chemotaxis and increase vascular permeability (Shao et al. 1998). Boswellic acid from *Boswellia serrata* also have inhibitory and apoptotic effect against the cellular growth of leukemia HL-60 cells (Huang et al. 2000). Clinical trials have demonstrated promising benefits from boswellic acids in rheumatoid arthritis, chronic colitis, ulcerative colitis, Crohn’s disease and bronchial asthma in addition to benefits for brain tumour patients. The effects of boswellic acid on central signaling pathways in human platelets and on various platelet functions have been investigated. It also caused a pronounced mobilization of Ca^2+^ from internal stores and induced the phosphorylation of p38 MAPK and elicits functional platelet responses (Poeckel et al. 2005). Boswellic acids have also been observed to inhibit human leukotriene elastase which may be involved in pathogenesis of emphysema. Human leukotriene elastase also stimulates mucus secretion and thus may play a role in cystic fibrosis, chronic bronchitis and acute respiratory distress syndrome (Rall et al. 1996; Safayhi et al. 1997). However, the clinical trials of gum-resin of *Boswellia serrata* have shown to improve symptoms in patients with osteoarthritis and rheumatoid arthritis (Poeckel and Werz 2006; Poeckel et al. 2006).

### Betulinic acid

Betulinic acid is a naturally occurring pentacyclic triterpene that exhibits a variety of biological activities including potent antiviral and anticancer effects (Alakurtti et al. 2006; Hsu et al. 2012). Mitochondria from cells, which were treated with betulinic acid, induced the cleavage of both caspase-3 and caspase-8 in cytosolic extracts. Cleavage of caspase-3 and 8 was preceded by disturbance of mitochondrial membrane potential and by generation of reactive oxygen species. Activation of caspase cascade was required for betulinic acid-triggered apoptosis. Interestingly, neuroblastoma cells resistant to doxorubicin-mediated apoptosis were still responsive to treatment with betulinic acid (Fulda et al. 1998; Fulda 2008). This revealed that betulinic acid inhibits the catalytic activity of topoisomerase I (Choudhary et al. 2002). Furthermore, betulinic acid exerts context dependent effects on cell cycle, it also reduces the expression of p21 protein in glioblastoma cells (Rieber and Strasberg Rieber 1998).

### β-Sitosterol

It is used to prevent and relieve prostate symptoms and has been tested for thousands of years in Asia and Mediterranean where the incidence of prostate problems—including prostate cancer—is considerably lower than that in the United States and Canada (Wilt et al. 1999; Richelle et al. 2004). Taking β-sitosterol at the dose of 60–110 mg/day significantly improve urinary symptoms. It increases the maximum urinary flow and decreases the volume of the urine left in the bladder. Like saw palmetto, β-sitosterol does not affect prostate size (Awad et al. 2000). β-Sitosterol is also used to lower cholesterol. It is an ingredient in the cholesterol-lowering margarine which is used to be known as take control (Berges et al. 2000). Aging is the main cause of enlarged prostate glands (Glynn et al. 1985). Testosterone is converted into another more powerful male hormone dihydrotestosterone in prostate cells. Dihydrotestosterone is responsible for triggering the division of prostate cells so their numbers increase by cell division process. Levels of dihydrotestosterone are known to be five times higher in enlarged prostate glands in those of normal sized prostate. If the conversion of testosterone to dihydrotestosterone is prevented, the BPH may not occur and may even be reversed once it has developed. β-Sitosterol works for enlarged prostate by inhibiting an enzyme called 5-α-reductase, blocking the conversion of testosterone into dihydrotestosterone, thereby decreasing the amount of dihydrotestosterone. Since, dihydrotestosterone is considered to be responsible for the enlargement of prostate, β-sitosterol helps to support normal prostate size. β-Sitosterol has been recommended by physicians for over 20 years as natural supplement to promote prostate health (Bent and Kane 2006). Based on the highly preliminary evidence, it has been suggested that β-sitosterol may also help strengthen the immune system (Pegel 1997). One study suggests that β-sitosterol can help prevent the temporary immune weakness that typically occurs during recovery from endurance exercise and can lead to post-race infections (Bouic et al. 1999). A randomized controlled trial of 47 patients with pulmonary tuberculosis investigated adjuvant β-sitosterol therapy vs placebo (Silveira e Sá et al. 2013). The β-sitosterol treatment group (average dose 60 mg/day) demonstrated increased weight gain, higher lymphocyte and eosinophil count and a generally faster clinical recovery (Donald et al. 1997).

### Ursolic acid

Mice fed with ursolic acid diet for 8 weeks delayed formation of prostate intraepithelial neoplasia. Similarly, mice fed with ursolic acid for 6 weeks inhibited progression of prostate intraepithelial neoplasia to adenocarcinoma as determined by hematoxylin and eosin staining. With respect to the molecular mechanism, it was observed that ursolic acid down regulated the activation of various pro-inflammatory mediators including NF-κB, TNF-α and IL-6 (Shanmugum et al. 2012).

## Future prospects

The terpenoids are synthesized from two five-carbon building blocks, which are known as isoprenoid units. Based on the number of building blocks, terpenoids are grouped into several classes, such as monoterpenes (e.g. carvone, geraniol, d-limonene and peril alcohol), diterpenes (e.g. retinol and retinoic acid), triterpenes (e.g. betulinic acid, lupeol, oleanolic acid and ursolic acid) and tetraterpenes (e.g. α-carotene, β-carotene, lutein, and lycopene) (Thoppil and Bishayee 2011; Rabi and Bishayee 2009a; Withers and Keasling 2007). Terpenoids have been found to be useful for the treatment of various types of diseases and disorders viz, antimicrobial, antifungal, antiparasitic, antiviral, antihyperglycemic, antihypoglycemic, anti-inflammatory and immunomodulatory properties (Wagner and Elmadfa 2003; Shah et al. 2009; Sultana and Ata 2008).

The development of transgenic plants against biotic stress like as insects has been a major successful scientific approach, mirrored by practical success of a limited number of pest-resistant transgenic crops in various countries. In some well-developed countries, this has been a result of vocal opposition to plant transformation technology itself; but in many examples, in both developed and developing countries, it is more a case of great potential economic benefits not being sufficient to make the introduction of transformed varieties of crops commercially viable.

The development of zero-cannabinoid cannabis chemotype has provided crude drugs that will facilitate discernment of the pharmacological effects and contributions of different fractions. Breeding work has already resulted in chemotypes that produce 98 % of monoterpenes as myrcene or 77 % as d-limonene. Through selective breeding of high terpenoid and phytocannabinoid-specific chemotype, has thus become rational target that may lead to novel approaches in treatment of different types of diseases and disorders.

The terpenes play important roles in plant interactions, plant defenses and the other environmental stresses (Chen et al. 2011). To better understand the physiological and ecological roles of specific terpene synthase genes and enzymes research in various areas is required. Roles of specific terpenes or general roles of classes of terpene synthases, regulation of terpene synthesis, phylogeny of terpene synthases, must be examined thoroughly in plants and ideally in the natural environments of the plants that produce these terpenes. The terpene synthase gene’s manipulation and their expression in model and non-model plants will be critical at this end. Despite many discoveries about the functions of terpene synthase genes, the ongoing and future structural and biochemical investigations of terpene synthases will continue to be field of exciting new discoveries. At present, functional characterization and regulation of terpene synthases have been completed only for subsets of terpene synthase families, including *Arabidopsis*, grapevine and spruce. Similar to other plant species, the biochemical functions and phylogeny of various number of terpene synthase proteins have already been established and known; there are only few experimentally determined three-dimensional structures of terpene synthases of plant origin (Gennadios et al. 2009; Kampranis et al. 2007). At the same time, large transcriptome sequencing projects targeted at plants species that produce interesting medicinally important metabolites will enhance the identification of comprehensive sets of terpene synthase genes in a large variety of non-model systems. An improved and updated knowledge on regulation of terpene metabolism and phylogeny of terpene synthases will facilitate the manipulation of terpene biosynthetic pathways for improvement of agronomic traits, biotransformation of medicinally important terpenes, floral scents (Lücker et al. 2001), plant defense against pests and pathogens (Schnee et al. 2006) and production of known and novel phytocompounds (Bohlmann and Keeling 2008).
